# When could human challenge trials be deployed to combat emerging infectious diseases? Lessons from the case of a Zika virus human challenge trial

**DOI:** 10.1186/s13063-019-3843-0

**Published:** 2019-12-19

**Authors:** Ricardo Palacios, Seema K. Shah

**Affiliations:** 1 Division of Clinical Trials and Pharmacovigilance, Instituto Butantan, São Paulo, SP Brazil; 2 School of Philosophy, Literature and Human Sciences, University of São Paulo, São Paulo, SP Brazil; 3 Mary Ann & J. Milburn Smith Child Health Research, Outreach, and Advocacy Center, Stanley Manne Children’s Research Institute, Ann & Robert H. Lurie Children’s Hospital of Chicago, Chicago, IL USA; 4 Department of Pediatrics, Northwestern University Feinberg School of Medicine, Chicago, IL USA

**Keywords:** Human challenge trials, Research ethics, Ethics in emergencies, Zika virus

## Abstract

Human challenge trials (HCTs) deliberately infect participants in order to test vaccines and treatments in a controlled setting, rather than enrolling individuals with natural exposure to a disease. HCTs are therefore potentially powerful tools to prepare for future outbreaks of emerging infectious diseases. Yet when an infectious disease is emerging, there is often substantial risk and uncertainty about its complications, and few available interventions, making an HCT ethically complex. In light of the need to consider ethical issues proactively as a part of epidemic preparedness, we use the case of a Zika virus HCT to explore whether and when HCTs might be ethically justified to combat emerging infectious diseases. We conclude that emerging infectious diseases could be appropriate candidates for HCTs and we identify relevant considerations and provide a case example to illustrate when they might be ethically acceptable.

## Background

Human challenge trials (HCTs), or controlled human infection models (CHIMs), involve deliberately exposing human participants to diseases to learn more about the early stages or transmission of a disease or to accelerate testing of interventions. One prominent example was the yellow fever experiments conducted by Walter Reed and colleagues in the early 1900s that proved that yellow fever was transmitted by mosquitoes [1]. Over the last few decades, controlled human malaria infection studies have enrolled approximately 2000 participants, with no serious adverse events or hospitalizations [2, 3]. Despite many important scientific advances from HCTs on cholera, dengue, influenza, and typhoid, some studies have been highly controversial [4], or even “ethically impossible,” such as the sexually transmitted infection experiments conducted in Guatemala in the 1950s [5]. Prominent ethical guidelines also preclude conducting HCTs on diseases that can cause serious morbidity or mortality and have no proven effective treatment, such as Ebola or Anthrax [6].

At first blush, HCTs seem ideal to prepare for future infectious diseases epidemics. Consider that the Coalition for Epidemic Preparedness Innovations (CEPI) was recently created in order to “finance and coordinate the development of new vaccines to prevent and contain infectious disease epidemics” [7]. For epidemics that loom on the horizon, developing vaccines without being able to test them on populations who are naturally exposed to a disease is difficult. Because HCTs involve isolating strains of a disease and deliberately infecting participants with the disease in a controlled environment, they make it possible to study interventions for infectious diseases in advance of outbreaks.

High priority diseases for epidemic preparedness include Nipah virus, MERS-CoV, Lassa virus, Ebola virus disease, Marburg virus disease, Zika virus, and Rift Valley Fever [8]. These are serious diseases that can be fatal, lack proven effective treatments, and have unknown and potentially long-term complications. Interestingly enough, the World Health Organization (WHO) has also acknowledged their predictions of diseases most likely to emerge as epidemics may not be accurate by including a “Disease X” in their priority list; Disease X “represents the knowledge that a serious international epidemic could be caused by a pathogen currently unknown to cause human disease” [8]. Notably, in 2013, Zika virus was a pathogen thought to cause only mild disease in humans and was not seen as a disease that could lead to a devastating epidemic.

In this paper, we consider the case of a Zika virus HCT as a springboard to determine whether and when an HCT of an emerging infectious disease could be permissible. Through this analysis, we isolate several conditions that might make it ethically acceptable to conduct an HCT on an emerging infectious disease. Recognizing that, for many high priority diseases, an HCT would expose participants to such high risk that it might not be ethically acceptable, we conclude by providing an illustrative case involving a potential outbreak of a Disease X and considering how an HCT might be useful in this scenario.

### The case of a Zika virus HCT

In 2015, Zika virus emerged as a major public health crisis in Brazil and other South American and Caribbean countries. Although the WHO declared the state of emergency surrounding Zika virus over in November 2016, it indicated that Zika is likely to be a persistent and unpredictable public health threat for years to come [9]. Some estimates indicate that Zika virus is asymptomatic in the vast majority of people infected and most of the symptomatic cases are mild and transient, but it can have potentially devastating consequences. In adults, Zika virus can cause neurological complications, including but not limited to Guillain–Barré syndrome, which is an acute, progressive neuropathy that can result in paralysis [10, 11]. It can also cause cardiac complications in adults, although some complications may emerge in the longer-term and more evidence is needed [12]. Perhaps of greatest concern, transmission of Zika virus from a pregnant woman to a fetus can cause congenital Zika syndrome. Congenital Zika syndrome typically involves microcephaly and potentially significant abnormalities in neurological development, visual impairment, cardiac defects, and other complications [13, 14]. Zika virus is known to be transmissible through mosquito vectors, sexual transmission, and mother-to-child transmission throughout pregnancy, but may also be transmitted in other ways [15, 16]. Until recently, the duration of infectivity for Zika virus was highly uncertain but is now thought to be transmissible for approximately 30 days after infection [15].

In 2016, researchers proposed conducting an HCT in which healthy volunteers would be intentionally exposed to Zika virus, which was a newly emerging public health threat at the time. Although the protocol for this trial is not publicly available, some of the details are widely known. The Zika virus HCT was proposed to learn more about the early stages of Zika infection and efficiently test whether vaccines can protect against Zika infection. The trial was to be conducted in the United States and enroll healthy volunteers who would not otherwise be exposed to Zika virus. Previous exposure to other viruses in the same family (flaviviruses such as dengue, yellow fever, West Nile) would have been an exclusion criterion for the study since there is a theoretical possibility of enhancement of disease in patients with antibodies from other flaviviruses. Participants would be confined in clinics for approximately two weeks [17, 18].

As the potential funders of such a trial, the National Institute of Allergy and Infectious Diseases and the Walter Reed Army Institute of Research were concerned that Zika virus HCTs were ethically complex. They therefore assembled an independent, multidisciplinary expert panel to address the question of whether a Zika virus HCT could be ethically justified, and if so, under what conditions. The panel included ethicists with expertise in several subfields of research ethics (i.e. the ethics of human challenge trials, study design, translational and early phase research, and research with pregnant women), a neurologist, two obstetrician/gynecologists, and an infectious disease physician. Panel members were vetted to ensure there were no conflicts of interest. The panel was charged with making recommendations to NIH about the ethical considerations for Zika HCTs in general. Unlike standard ethics review committee or institutional review board processes, the panel members did not review a study protocol. The panel’s deliberations were not meant to supplant existing review processes, but to supplement them. Accordingly, the panel considered the latest scientific and epidemiological information about Zika virus, existing ethical frameworks for HCTs, and ongoing research into Zika vaccines.

The panel held several teleconferences and one in-person meeting with presentations from experts from a variety of backgrounds. After extensive deliberation, the panel drafted a report that was released in February 2017. The Zika HCT ethics expert panel offered a preliminary ethical framework for Zika HCTs and concluded that Zika virus HCTs could be ethically justified in principle but would be premature at the time [18]. The two main reasons that the panel concluded a Zika HCT would be premature were that: (1) there was potential risk to bystanders outside of the study who had not given their consent to participation and not enough was known about Zika transmission to ensure they could be protected; and (2) existing studies were ongoing and the panel could not be confident that a Zika HCT was needed to accelerate the course of vaccine development. First, although Zika virus appears to be asymptomatic for most of those who are infected, exposure to fetuses can be catastrophic [13, 14]. At the time of the panel’s review, it was not known how long participants might be capable of sexual transmission to others. Upper estimates were that participants could be infectious for six months, making it difficult to ensure that adequate precautions could be taken to avoid transmission to fetuses. Second, since information was already being generated about vaccine safety and efficacy through ongoing research, it was unclear at the time whether a Zika HCT offered sufficient potential for societal benefit to justify the risk.

Although these were the most critical and unresolved issues facing Zika HCTs at the time, the panel also noted several other important ethical considerations, including ensuring that there was a robust informed consent process, sufficient but not undue compensation for research participation, a plan to respect the right to withdraw, a system for compensation for research-related injury, and consultation with the community in which a Zika HCT would be conducted—considerations which may be helpful for HCTs more generally [18].

It is important to note that external conditions, the evidence available about Zika virus, and the epidemiology of Zika virus have changed since the panel submitted its recommendations. The period of infectivity for those infected with Zika virus is much better known; Zika virus can be transmitted for roughly 30 days in individuals whose viral loads are not high [19] (rather than six months, which was the previous recommendation for the amount of time Zika-infected individuals should avoid unprotected sexual activity) [20]. Additionally, although a phase II Zika vaccine trial is ongoing, enrollment is lower than projected, a phase III trial to test efficacy does not seem feasible, and some drug and vaccine developers have halted research on Zika virus [21]. This suggests that a Zika HCT could have clear and considerable value if conducted today, provided that there was independent, rigorous review of both the ethical considerations involved and a plan to protect bystanders and research participants.

### Application of the Zika HCT case to other HCTs on emerging infectious diseases

Closer examination of the two major issues that arose in the Zika HCT ethics consultation provides lessons for other potential HCTs on emerging infectious diseases. At the time of the initial consultation, the panel focused on the: (1) crowded field of interventions and ongoing studies and uncertain social value; and (2) potentially high and uncertain risk to participants and third parties if the trial was conducted in the United States.

First, as was the case with Zika virus, many stakeholders are likely to be interested in addressing an emerging infectious disease in the midst of an outbreak, making it difficult to determine how much value an HCT would add. Within a year of the panel’s report, it became clear that an efficacy trial would be extremely difficult to conduct giving the declining numbers and unpredictability of Zika outbreaks, and the initial uncertainty about the value of a Zika HCT was resolved [17]. One possibility, then, is that riskier HCTs of emerging infectious diseases may not be part of the first-line response to an epidemic, but potentially valuable tools in the arsenal that could be deployed when other possibilities have been exhausted.

Second, as previously mentioned, emerging infectious diseases that are prioritized have potentially serious complications and are often characterized by high uncertainty. For example, the modes and duration of transmission of Zika virus were not known at the time of the review of the ethics of Zika HCTs, so the risks of transmission to people outside of the study were difficult to determine, let alone mitigate. As has been described elsewhere, the Zika HCT ethics panel struggled with the question of how to justify uncertain risks to bystanders. Third parties outside of the research may not know or give consent to being exposed to risk of infection with Zika virus, and there is limited guidance on how to address risk to research bystanders [18, 22]. We have written elsewhere about this challenge [22] and will not belabor this further, but will focus on whether there is or should be an upper limit of risk for research participants here.

There is limited regulatory and ethical guidance on whether there is an upper limit of allowable risk in research. The Nuremberg Code was one of the earliest codes of research ethics and addresses this issue directly, stating that “No experiment should be conducted where there is an a priori reason to believe that death or disabling injury will occur; except, perhaps, in those experiments where the experimental physicians also serve as subjects” [23]. Relevant for our purposes, this exception was included in order not to condemn the yellow fever HCTs conducted by Walter Reed and colleagues, in which members of the research team were enrolled, and one investigator died from yellow fever. However, because the Nuremberg Code’s statement does not provide probabilities to indicate what risk of death or serious injury would be unacceptable, it might inadvertently rule out many other studies that are commonly considered acceptable today, such as phase I studies with healthy volunteers that have a very low risk of death and disabling injury [24–26]. It is also unclear whether self-experimentation is a secure protection against excessive risk, since power dynamics might make it difficult for junior investigators to decide not to participate in a study run by their superiors. Furthermore, it is unclear why the fact that a scientist may choose to sacrifice her short-term interests for research she believes in can justify exposing others to risk. Her judgment may be subject to bias about the importance of her own research [27]. The Nuremberg Code’s language about acceptable risk in research has not been adopted by prominent codes of ethics that are in widespread use today, possibly for these reasons.

For example, the U.S. Federal regulations merely state that risks must be minimized and justified by the benefits to society, suggesting that any level of risk could be permissible as long as there was sufficient social value and informed consent by the participants [28]. The most recent update to the international ethics guidelines promulgated by the Council for International Organizations of the Medical Sciences also simply states in its guidelines that risks should be minimized and justified by the benefits to society; however, the commentary underlying this statement indicates that there is some upper level of risk in research that cannot be justified. Notably, CIOMS draws this line of unacceptable risk by ruling out examples of research with extraordinary risks. The examples CIOMS provides of an unacceptable study are of HCTs: “[A] study that involves deliberately infecting healthy individuals with anthrax or Ebola - both of which pose a very high mortality risk due to the absence of effective treatments - would not be acceptable even if it could result in developing an effective vaccine against these diseases” [6]. This language seems to suggest that HCTs on many high-priority emerging infectious diseases could not be justified because of the high level of risk to participants.

In contrast, others have not ruled out HCTs on emerging infectious diseases and noted that the way risks should be calculated in the context of an ongoing outbreak might be relevant. Joffe and Miller argue that:

[V] olunteers for high-risk public-health research may be subject to substantial background risk from public-health threats, such as potentially lethal infectious diseases for which effective treatment is lacking. It is thus the incremental net risk from the research that must be assessed against the prospect of public-health benefits from the study results [6].

Joffe and Miller give the example of an epidemic of avian influenza and note that, just as firefighters or rescue workers facing a large-scale disaster take on higher risks than they would in the ordinary course, so too could research participants [6].

Accordingly, another possible way an HCT on an emerging infectious disease could be ethically acceptable is if the HCT was conducted in a setting where an outbreak was occurring. In the context of HCTs, this is typically referred to as an “endemic setting,” but perhaps a clearer way to describe it is that the participants should be drawn from a group already facing some background risk of infection. This may be a way of reducing the net risk to which research volunteers are exposed, since they are already at some level of risk of having the disease, and the difference is that they now will have near-certainty of being infected.

There are potential drawbacks to conducting HCTs in endemic settings, however, but these can be addressed in some cases [29]. First, there may be issues of scientific integrity because it can be very difficult to exclude the possibility that participants were previously infected or are currently infected before enrollment. Although these challenges will make some HCTs in endemic settings problematic, enrolling participants with prior exposure can be scientifically beneficial because it may help devise an intervention that is more responsive to the needs of people in endemic settings. Additionally, in other cases, there may be groups of people within the population who can be reliably tested to ensure they do not have previous exposure. Second, in some locations with high potential for the disease to spread and it is difficult to prevent transmission to others, the risk to third parties will also have to be weighed in the balance. Although Zika virus is asymptomatic in roughly half to three-quarters of those who are infected [30], exposure to fetuses can be catastrophic [31]. If an emerging infectious disease was not transmissible to fetuses, then, an HCT of an emerging infectious disease might be easier to justify. Similarly, if a disease was not sexually transmitted and only transmitted by mosquitoes, or if the length of time in which the disease could be transmitted was of a certain and relatively short duration, the risk to third parties could more easily be addressed.

In sum, if a population could be identified who could give consent and had a likelihood of being largely unaffected by the disease, researchers could be confident that the disease would not spread outside of the research participants, and if the research could be conducted in a setting with a relatively high background risk of infection and careful monitoring throughout the study, then an HCT on an emerging infectious disease could be relatively straightforward to justify ethically (provided of course that standard research ethics criteria were satisfied as well).

### Application to a “Disease X”

Although Zika virus was discovered in Uganda in 1947, it did not raise international concern until relatively recently. In 1956, Bearcroft inoculated himself with Zika virus and noted only very mild, transient symptoms [32]. Due to a number of factors, such as modification in land use and livestock patterns, interactions between humans and wildlife, climate, and globalization, viruses can acquire unexpected pathogenic features [33], as seems to have occurred in recent years with Zika virus. Recognizing that the world was not prepared for the most recent Zika epidemic, preparedness for the next “Disease X” is critically important.

**Fig. 1:**
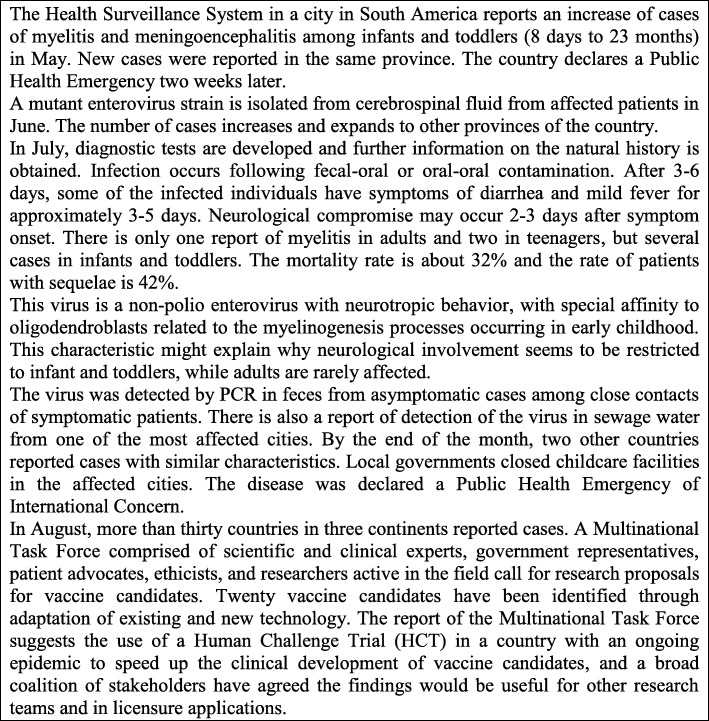
Example of a potential Disease X: mutant enterovirus associated to neurovirulence

We use a case of a hypothetical neurovirulent non-polio enterovirus to illustrate a possible use of HCT with an emerging infectious disease; this is one way to flesh out the WHO’s description of a “Disease X” that might also be ethically acceptable to study in an HCT (Fig. 1) (Additional file 1).

Provided that the general conditions for ethical research are met [34], this example illustrates a scenario in which an HCT on an emerging infectious disease might be relatively straightforward to justify ethically because the conditions outlined above are present: (1) there is a population—adults—who are likely not to have serious complications from infection with Disease X; (2) there is a possibility of conducting the HCT in a setting where the outbreak is ongoing, thereby lowering the net risk to which participants are exposed; (3) there is broad agreement among stakeholders about the purpose of the HCT and willingness to use the results to accelerate vaccine development; and (4) the modes of transmission (i.e. fecal and oral) can be more easily monitored to ensure that volunteers in the research do not spread the disease to bystanders. Additionally, it would be important that data from an adult HCT will be relevant to children, who are the target population for the vaccine, and that the research can be conducted in a safe and scientifically sound manner in an endemic setting.

As with the example of the Zika HCT, there are several other issues that should be addressed for an HCT on Disease X to be ethically acceptable. Although we lack the space to address them fully, we will touch on two critical issues here. First, the level of payment would have to be sufficient to compensate participants for the amount of time they would contribute to the study and could be relatively high if participants had to be confined to avoid transmission to others for an extended period of time. This may raise concerns about undue inducement—more specifically, that participants might ignore the risks involved [35]—or lie or withhold disqualifying information in order to participate [36]. Existing data suggest that high payments do not necessarily compromise HCT participants’ ability to understand the risks [37–39], and some studies suggest that participants who are motivated by money pay more attention to the risks [40–43], perhaps recognizing that elevated risk is a reason for high payments. To address concerns about understanding of risks, researchers could try to improve understanding by using rigorous informed consent processes that allow extra time for processing of information and discussion, or tests of understanding with feedback for incorrect answers [44]. To address concerns about withholding information, researchers could ensure that inclusion/exclusion criteria are not known to participants in advance so they will not know what information is important to enroll in the research. Additionally, researchers could ensure they have ways to objectively verify all inclusion/exclusion criteria that are important for safety reasons or scientific validity, instead of depending on what participants report. Researchers should consider the general context in the recruitment areas to determine a reasonable amount of compensation, which may be especially difficult to determine in marginalized communities. Local ethics committees can provide valuable input based on their knowledge of the community.

Second, there might be justice concerns that the data from an HCT of Disease X would not be used to generate benefits that would be valuable to those who are most affected by Disease X. For an HCT on Disease X to have sufficient social value to justify exposing individuals to risk, it is unlikely that producing a vaccine aimed at travelers rather than those in endemic regions would be sufficient to motivate the study. To the extent that the potential global social value is what justifies conducting the research, researchers and sponsors should endeavor to make the benefits of their research broadly available [45].

Even if the conditions outlined above were not able to be met initially, an HCT might still be an ethically acceptable response down the line. As more information was gathered about the disease and it became difficult to conduct research in the field to license a vaccine, the social value of an HCT would increase.

Finally, it is worth noting that the scientific production during a public health emergency is overwhelming. The number of articles on Zika virus registered in the National Center for Biotechnology Information’s PubMed database was 81 between 1952 and 2013, 26 in 2014, 38 in 2015, 1735 in 2016, 1855 in 2017, and 5480 by the beginning of April 2018. Although this can be a potential challenge for ethics review processes to sort the wheat from the chaff, it is also another reason that waiting to conduct an HCT for a relatively short period could result in much more information being available to conduct the HCT safely and in a more targeted fashion.

## Conclusion

Examining the case of a potential Zika virus HCT reveals that HCTs on emerging infectious diseases could be ethically acceptable under the right conditions. A more comprehensive framework and rigorous review processes are urgently needed [21], particularly if a higher risk HCT is proposed in the setting of a dire emergency. In the meantime, these cases illustrate that preparedness for emerging diseases should include rigorous consideration of ethical issues in advance of future outbreaks. Advance ethical preparation is critical in order to avoid wasting scarce resources and maintaining public trust in outbreaks demanding rapid and effective responses to safeguard the public’s health.

## Supplementary information

**Additional file 1.** Reviewers Report

## Data Availability

Not applicable.

## Abbreviations

CEPI: Coalition for Epidemic Preparedness Innovations
CHIM: Controlled human infection models
CIOMS: Council for International Organizations of Medical Sciences
HCT: Human challenge trial
NIH: United States National Institutes of Health
WHO: World Health Organization
