# Supplementary material for: When could human challenge trials be deployed to combat emerging infectious diseases? Lessons from the case of a Zika virus human challenge trial
Source: Trials. 2019 Dec 19;20(Suppl 2):702. doi: 10.1186/s13063-019-3843-0 (PMC6921433; doi:10.1186/s13063-019-3843-0)
Supplement: Supplementary file 1 — Additional file 1. Reviewers Report [file 13063_2019_3843_MOESM1_ESM.docx]

**Reviewer’s Report**

**Article Title: When could human challenge trials be deployed to combat emerging infectious diseases? Lessons from the case of a Zika virus human challenge trial.**

**Date: 1^st^ February 2019**

**Reviewer name: Jimmy Whitworth**

**Reviewer’s report:**

This is an interesting and useful commentary piece. My comments are relatively minor suggestions.

The authors frame the ethical issues well, but I think it would be worth discussing the benefit/harm balance at the individual level as well as at societal level. The issue of undue inducement could be explored further too. Presumably considerable compensation would be needed in order to recruit participants willing to be confined to a clinic for two weeks for an infection which is of no risk to them. It might also be worth mentioning the issues of distributive justice and whether the proposed vaccine would be aimed primarily at travellers or those residing in endemic/epidemic zones. I am not sure quite what is meant by a community engagement approach – line 4 page 6. I would have thought that would be important to conduct some exploratory studies in target communities to gauge whether it would be possible to recruit sufficient participants into the proposed study.

I have concerns about the suggestion of conducting HCTs in areas where natural transmission is occurring. This might be ethically desirable as it reduces the absolute increased risk to participants, but it makes the study itself logistically and analytically harder to conduct as it will be very difficult to exclude the possibility that participants are already incubating natural infection when recruited to the study. This is why malaria HCTs are usually conducted outside of malaria endemic areas.

I also had a concern about the enterovirus scenario. The-at risk population is infants and toddlers, and yet I think the proposed target group for the vaccine trial is adults. This is not ideal, as there could be important differences in response to the vaccine between adults and infants. This problem should be acknowledged.

The sentence starting ‘It is worth noting….’ Line 13, page 12 comes as a surprise and does not follow from the previous text. This should probably be the start of a new paragraph.

References 16 and 20 are duplicates.

**Level of interest. Please indicate how interesting you found the manuscript:**

This is interesting and provides some challenging concepts and information.

**Quality of written English. Please indicate the quality of language in the manuscript:**

Good

**Quality of figures; All images and figures within the manuscript should be genuine i.e. without evidence of manipulation. No specific feature within an image may be enhanced, obscured, moved, removed, or introduced. If you have concerns about the veracity of the figure, please comment below.**

No concerns

**Statistical review**

Is it essential that this manuscript is seen by an expert statistician? If so, please give your reasons in your report above. No

**Declaration of competing interests**

Please complete a declaration of competing interests, considering the following questions:

1. Have you in the past five years received reimbursements, fees, funding, or salary from an organisation that may in any way gain or lose financially from the publication of this manuscript, either now or in the future?

2. Do you hold any stocks or shares in an organisation that may in any way gain or lose financially from the publication of this manuscript, either now or in the future?

3. Do you hold or are you currently applying for any patents relating to the content of the manuscript?

4. Have you received reimbursements, fees, funding, or salary from an organization that holds or has applied for patents relating to the content of the manuscript?

5. Do you have any other financial competing interests?

6. Do you have any non-financial competing interests in relation to this paper?

If you can answer no to all of the above, write 'I declare that I have no competing interests' below. If your reply is yes to any, please give details below.

I declare I have no competing interests.

**Open Peer Review Declaration**

I agree to the open peer review policy of the journal. I understand that my name will be included on my report to the authors and, if the manuscript is accepted for publication, my named report including any attachments. I upload will be posted on the website along with the authors' responses. I agree for my report to be made available under an Open Access Creative Commons CC-BY license (http://creativecommons.org/licenses/by/4.0/). I understand that any comments which I do not wish to be included in my named report can be included as confidential comments to the editors, which will not be published.

J Whitworth

**Were you mentored through this peer review?**

No

**Reviewer’s Report**

**Article Title: When could human challenge trials be deployed to combat emerging infectious diseases? Lessons from the case of a Zika virus human challenge trial**

**Date: 2/20/19**

**Reviewer name: Sally Hunsberger**

**Reviewer’s report):**

This article discusses human challenge trials and when they are justified and when they are not appropriate. The authors use Zika as a case study.

The following important article should be referenced. Vannice, Kirsten S., et al. "Demonstrating vaccine effectiveness during a waning epidemic: A WHO/NIH meeting report on approaches to development and licensure of Zika vaccine candidates." *Vaccine* (2019).

Reference 16 and 20 are the same reference.

In the background section the authors should not only mention that HCT’s have been used for Malaria but for Dengue and influenza to show broader use.

In background the authors should also note viruses like HIV and TB that are not possible to study in a HCT. This is a different point than the last sentence where people ran an unethical HCT.

The sentence “The severity of and uncertainty associated with these disease makes it very difficult to justify deliberately exposing research participants to these diseases in an HCT.” On page 3 lines 5-6, seems out of place in this paragraph and disrupts the flow. The rest of the paragraph is describing high priority diseases and what they are.

The following sentence on page 4 line 2-5, “Some estimates indicate that Zika virus is asymptomatic in the vast majority of people infected, but it has potentially devastating consequences, including congenital Zika syndrome in infants, and Guillain-Barré syndrome and cardiac complications in adults”. Needs to be fleshed out a bit more. Need to emphasize that the congenital Zika syndrome in infants is through transmission and transmission is a huge issue in HCT. Also, the authors should say that there may be neurologic and cardiac LATE term consequences for symptomatic and asymptomatic people infected with Zika. Again, since this is still a question it impacts on the ethics of dong a HCT.

Page 7 line 9. “….may not BE part…” missing the BE.

Page 10 lines 15-20 are very important and are a very nice summary of the challenges with HCT with zika. I think it would help the reader if this information could be moved to the beginning of the article or at least the beginning of the section on zika starting on page 3 line 21.

**Level of interest. Please indicate how interesting you found the manuscript:**

>HCT’s are important and need to be discussed. The wider scientific community often does not think about them.

**Quality of written English. Please indicate the quality of language in the manuscript:**

There were some very long sentence that include several points. The writing would be clearer if the sentences were targeted and made one point.

**Quality of figures; All images and figures within the manuscript should be genuine i.e. without evidence of manipulation. No specific feature within an image may be enhanced, obscured, moved, removed, or introduced. If you have concerns about the veracity of the figure, please comment below.**

**Statistical review**

Is it essential that this manuscript is seen by an expert statistician? If so, please give your reasons in your report above.

I am a statistician reviewing this.

**Declaration of competing interests**

Please complete a declaration of competing interests, considering the following questions:

1. Have you in the past five years received reimbursements, fees, funding, or salary from an organisation that may in any way gain or lose financially from the publication of this manuscript, either now or in the future? No.

2. Do you hold any stocks or shares in an organisation that may in any way gain or lose financially from the publication of this manuscript, either now or in the future? No

3. Do you hold or are you currently applying for any patents relating to the content of the manuscript? No

4. Have you received reimbursements, fees, funding, or salary from an organization that holds or has applied for patents relating to the content of the manuscript? No

5. Do you have any other financial competing interests? No

6. Do you have any non-financial competing interests in relation to this paper? No

If you can answer no to all of the above, write 'I declare that I have no competing interests' below. If your reply is yes to any, please give details below. I declare that I have no competing interests.

**Open Peer Review Declaration**

I agree to the open peer review policy of the journal. I understand that my name will be included on my report to the authors and, if the manuscript is accepted for publication, my named report including any attachments. I upload will be posted on the website along with the authors' responses. I agree for my report to be made available under an Open Access Creative Commons CC-BY license (http://creativecommons.org/licenses/by/4.0/). I understand that any comments which I do not wish to be included in my named report can be included as confidential comments to the editors, which will not be published.

> Sally Hunsberger

**Were you mentored through this peer review?**

> No

**Authors’ responses to reviewers: When could human challenge trials be deployed to combat emerging infectious diseases? Lessons from the case of a Zika virus human challenge trial**

**Date:** 5 April 2019

Dear Editors and Reviewers:

We thank the reviewers for their kind words about our manuscript and the helpful comments they have provided. We provide point-by-point responses to both reviewers’ comments below.

1. **Reviewer name: Jimmy Whitworth**

Reviewer’s comments **(in bold)** and author responses *(in italics)***:**

- **This is an interesting and useful commentary piece. My comments are relatively minor suggestions. The authors frame the ethical issues well, but I think it would be worth discussing the benefit/harm balance at the individual level as well as at societal level.**

*Thank you for this helpful suggestion. We have added language about this on page 5 in our discussion of a Zika HCT, and have also tried to make this balance more clear in the section on whether there is an upper limit of risk in research on page 9.*

- **The issue of undue inducement could be explored further too. Presumably considerable compensation would be needed in order to recruit participants willing to be confined to a clinic for two weeks for an infection which is of no risk to them.**

*We appreciate this suggestion, and admittedly were struggling to describe the Zika report’s analysis without recapitulating it entirely. As such, we have not added more about undue inducement in describing the case of a Zika HCT, but have added much more discussion of this issue when discussing the case of an HCT on Disease X.*

- **It might also be worth mentioning the issues of distributive justice and whether the proposed vaccine would be aimed primarily at travellers or those residing in endemic/epidemic zones.**

*This is a great point, and we have added language to address this in the example of an HCT on Disease X as well.*

- **I am not sure quite what is meant by a community engagement approach – line 4 page 6. I would have thought that would be important to conduct some exploratory studies in target communities to gauge whether it would be possible to recruit sufficient participants into the proposed study.**

*Thanks for the opportunity to clarify this point. In the Zika report, we indicated that consultation with the community in which a Zika HCT would be conducted could be valuable, and have now included similar language in this manuscript to elucidate this point. There are several different ways in which community engagement could be helpful, one of which would be to conduct feasibility assessments in relevant communities, as the reviewer notes. One of us has a paper currently under submission about the many different ways in which community engagement could be helpful for addressing third party risks in human challenge trials, so we know there is a lot to say here, but we lack the space to address this adequately in this manuscript.*

- **I have concerns about the suggestion of conducting HCTs in areas where natural transmission is occurring. This might be ethically desirable as it reduces the absolute increased risk to participants, but it makes the study itself logistically and analytically harder to conduct as it will be very difficult to exclude the possibility that participants are already incubating natural infection when recruited to the study. This is why malaria HCTs are usually conducted outside of malaria endemic areas.**

*We appreciate this point and have now included more discussion of the drawbacks of conducting HCTs in endemic settings. As the reviewer is likely aware, malaria HCTs are increasingly being conducted in endemic settings, and part of the justification is that a history of exposure might be relevant for designing a vaccine that is more effective in endemic settings. The manuscript now makes clear that there are important benefits and risks to consider in making the decision of where to conduct an HCT.*

- **I also had a concern about the enterovirus scenario. The-at risk population is infants and toddlers, and yet I think the proposed target group for the vaccine trial is adults. This is not ideal, as there could be important differences in response to the vaccine between adults and infants. This problem should be acknowledged.**

*This is a great point, and we have acknowledged the issue but unfortunately lack the space to do much more than that. As this is a hypothetical scenario, we are assuming something can be learned from adults that is relevant to children, but that it may not be sufficient to do an HCT in adults alone in order to develop a vaccine for the target population.*

- **The sentence starting ‘It is worth noting….’ Line 13, page 12 comes as a surprise and does not follow from the previous text. This should probably be the start of a new paragraph.**

*We agree with this suggestion and have made this sentence the start of a new paragraph.*

- **References 16 and 20 are duplicates.**

*Thanks for catching this; we have added a new reference suggested by the second reviewer for note 20 that is a better source for the same point.*

2. **Reviewer name: Sally Hunsberger**

Reviewer’s comments **(in bold)** and author responses *(in italics)***:**

- **The following important article should be referenced. Vannice, Kirsten S., et al. "Demonstrating vaccine effectiveness during a waning epidemic: A WHO/NIH meeting report on approaches to development and licensure of Zika vaccine candidates." *Vaccine* (2019).**

*We agree it is important to include this reference. We have actually used this in place of previous reference 20 now, as we think it is a better reference for the same point.*

- **Reference 16 and 20 are the same reference.**

*See above.*

- **In the background section the authors should not only mention that HCT’s have been used for Malaria but for Dengue and influenza to show broader use.**

*That’s exactly right; we have added this information.*

- **In background the authors should also note viruses like HIV and TB that are not possible to study in a HCT. This is a different point than the last sentence where people ran an unethical HCT.**

*We appreciate the importance of stating the limits of what would be allowed in an HCT, and have included some indication of HCTs that might be out of bounds even in theory by referencing the prohibition on HCTs on Anthrax or Ebola in the Council for International Organizations on Medical Sciences. Although Miller & Grady have argued that an HCT on HIV would not be ethically acceptable, we know others have argued that an HCT with HIV could be acceptable depending on strain selection and whether a cure is available. Additionally, we have heard that an HCT on TB is currently under development, though it is worth noting that the challenge strain might involve administration of BCG vaccine.*

- **The sentence “The severity of and uncertainty associated with these disease makes it very difficult to justify deliberately exposing research participants to these diseases in an HCT.” On page 3 lines 5-6, seems out of place in this paragraph and disrupts the flow. The rest of the paragraph is describing high priority diseases and what they are.**

*This is an excellent point. We have edited and moved the sentence to the next paragraph to explain why we use Disease X as our example of an HCT on a high priority emerging infectious disease.*

- **The following sentence on page 4 line 2-5, “Some estimates indicate that Zika virus is asymptomatic in the vast majority of people infected, but it has potentially devastating consequences, including congenital Zika syndrome in infants, and Guillain-Barré syndrome and cardiac complications in adults”. Needs to be fleshed out a bit more. Need to emphasize that the congenital Zika syndrome in infants is through transmission and transmission is a huge issue in HCT. Also, the authors should say that there may be neurologic and cardiac LATE term consequences for symptomatic and asymptomatic people infected with Zika. Again, since this is still a question it impacts on the ethics of dong a HCT.**

*We agree this information is highly relevant to the ethics of conducting a Zika virus HCT, and have fleshed this out much more in the current draft. We were not certain whether the “late term” comment was in reference to transmission late in pregnancy or longer-term effects, but now address both possibilities.*

- **Page 7 line 9. “….may not BE part…” missing the BE.**

*Thanks for catching this typo; we have added the word “BE”.*

- **Page 10 lines 15-20 are very important and are a very nice summary of the challenges with HCT with zika. I think it would help the reader if this information could be moved to the beginning of the article or at least the beginning of the section on zika starting on page 3 line 21.**

*As we mentioned in a comment to the first reviewer, one challenge we struggled with in writing this paper is that we were trying not to reiterate the reasoning of the Zika HCT ethics panel. We now appreciate we should have provided more explanation of the ethical challenges associated with a Zika HCT so this paper can really stand alone for readers who have not read the report. We have moved this information to the section on a Zika HCT on page 5.*

- **There were some very long sentence that include several points. The writing would be clearer if the sentences were targeted and made one point.**

*Agreed. We have tried to shorten sentences throughout the manuscript to make the writing clearer and more cogent.*
